# Patient characteristics associated with differences in patients' evaluation of their general practitioner

**DOI:** 10.1186/1472-6963-8-178

**Published:** 2008-08-20

**Authors:** Hanne Nørgaard Heje, Peter Vedsted, Ineta Sokolowski, Frede Olesen

**Affiliations:** 1 The Research Unit for General Practice, University of Aarhus, Aarhus, Denmark

## Abstract

**Background:**

Knowledge of the extent to which patient characteristics are systematically associated with variation in patient evaluations will enable us to adjust for differences between practice populations and thereby compare GPs. Whether this is appropriate depends on the purpose for which the patient evaluation was conducted. Associations between evaluations and patient characteristics may reflect gaps in the quality of care or may be due to inherent characteristics of the patients. This study aimed to determine such associations in a setting with a comprehensive list system and gate-keeping.

**Methods:**

A nationwide Danish patient evaluation survey among voluntarily participating GPs using the EUROPEP questionnaire, which produced 28,260 patient evaluations (response rate 77.3%) of 365 GPs. In our analyses we compared the prevalence of positive evaluations in groups of patients.

**Results:**

We found a positive GP assessment to be strongly associated with increasing patient age and increasing frequency of attendance. Patients reporting a chronic condition were more positive, whereas a low self-rated health was strongly associated with less positive scores also after adjustment. The association between patient gender and assessment was weak and inconsistent and depended on the focus. We found no association either with the patients' educational level or with the duration of listing with the GP even after adjusting for patient characteristics.

**Conclusion:**

Adjustment for patient differences may produce a more fair comparison between GPs, but may also blur the assessment of GPs' ability to meet the needs of the populations actually served. On the other hand, adjusted results will enable us to describe the significance of specific patient characteristics to patients' experience of care.

## Background

Patient evaluations of care are increasingly being included in systematic assessments aimed at improving the quality of general practice. The extent to which patients are able to assess the technical quality of care is being debated [1,2], however, when the focus is on doctor-patient relationships, patients' experience is an obvious and valuable assessment tool [3,4]. Besides, WHO defines a high degree of patient satisfaction as one of five criteria for good health care quality [5].

Variation in patient evaluations of general practice reflects differences between the evaluated general practitioners (GPs) and their practices, and between the patients themselves.

A patient's evaluation is shaped by the actual contents and quality of the contacts over time, the patient's experience of the contact, his or her expectations, former experience, needs and the reason for the encounter. These aspects are also influenced by external factors such as family and friends, press and official (health) authorities and the cultural and historical setting at the time of the patient's life [6,7].

Knowledge of the extent to which patient characteristics are systematically associated with variation in patient evaluations will help to discover possible gaps in the quality of care and also enable us to adjust for differences between practice populations and thereby compare GPs. In addition, we will be able to calculate the variation attributable to GP factors. On the other hand adjustment for patient characteristics will blur the GPs' ability to tailor care according to the individual patient [8,9].

Many studies and reviews have explored associations between patients' evaluation of care and their characteristics. The most consistent finding is that older patients and patients with a low educational level rate care higher than younger patients and better educated patients [2,4,6,10-14]. While many studies find no gender difference in the assessment of the GP, a few studies report women to be more satisfied with care than men [4,10]. Similar results are reported regarding patients' socioeconomic status with a few studies reporting patients with a high socioeconomic status being a little more satisfied compared with less well off patients [6,11]. Diverging associations between patients' health and their care assessment have also been described [14].

Earlier studies have been carried out in different settings like hospital, outpatient clinics and general practice thus making comparisons irrelevant. The applied instruments have been of a varying quality and often study populations have often been small [15]. Some authors have set out to measure patients' satisfaction with care, although a proper theory for the construct "satisfaction" has never been worked out [1]. A European tool for patients' assessment of specific aspects of general practice care, the EUROPEP instrument, was developed through the 1990s [16,17] on the basis of patients' care priorities [18] and refined through a strict validation procedure [19]. The 23 items are displayed in [see Additional file 1].

Patients' assessment of specific aspects of care may be shaped by the context including the health care organisation which reduces the transferability of standards between organisations. The Danish health care system is based on self-employed GPs working as gatekeepers for the public health services on a contract basis serving patients on their lists (a brief introduction to the Danish general practice is given in [see Additional file 2]).

This study aimed to determine to which extent variations in patients' evaluation of the GPs were associated with the patients' gender, age, health, educational level, frequency of attendance and adherence to the GP in a setting with a comprehensive list system and gate-keeping.

## Methods

### Study population

In 2002–4 all 2181 GPs from ten Danish counties were invited to carry out patient evaluations of their practices. A total of 365 GPs (16–34% of all GPs in these counties) signed in. The participating GPs handed out questionnaires to 100 successive patients seen in the surgery or at home visits. The patients were at least 18 years of age, were listed in the practice and were able to read and write Danish. They were informed that their replies were anonymous to the doctor. Each questionnaire was identified by a serial number connecting it with the GP who handed it out and to the patient. If the GP had not handed out all questionnaires within two weeks, (s)he returned the rest to the project secretariat for registration.

The patients were asked to assess the GP they considered to be their personal GP based on their contact experience over the past 12 months. They were also asked to write the GP's name on the questionnaire to confirm which GP was assessed and to allow individual assessment of GPs in partnership practices. The questionnaires were returned by the patients in prepaid envelopes to the project secretariat.

In order to be able to carry out the reminder procedure the GPs registered the names, addresses and serial numbers from the questionnaires handed out. Reminders with new questionnaires were sent to non-responding patients three to five weeks after the GPs' distribution of the patient-questionnaires and the patient lists were thereafter destroyed.

### The questionnaire

The questionnaire contained the 23 items forming the EUROPEP instrument [16,17]. These questions covered specific aspects of general practice care and were grouped into five dimensions: doctor-patient relationship (6 questions), medical care (5 questions), information and support (4 questions), organisation of care (2 questions) and accessibility (6 questions). The answers were marked on a 5-point Likert scale ranging from "poor" to "excellent", with "acceptable" as the middle value. Alternatively, the patients could choose a sixth category "not able to answer/not relevant". The questionnaire also included questions about the patient's gender, age, educational level, frequency of attendance to a general practice for the previous 12 months, time listed with the GP, self-rated health and chronic conditions.

In the project secretariat we coded the diagnoses reported by patients with chronic conditions according to the major ICPC-2 groups [20] with the label K for cardiovascular, R for respiratory and T for endocrine diseases along with two ad hoc-groups labelled C for patients reporting cancer diagnoses and M for patients with multiple diagnoses.

### Assessments of the GPs

Within each dimension, a patients' evaluation was included only if 50% or more of the items had been answered in one of the six categories. An answer was considered positive if it fell in one of the two most favourable categories. The assessment of the dimension was categorised as 100%, 50–99% or 0–49% positive depending on how many of the items marked on the 5-point Likert scale were positive. We compared the prevalences of assessments in the 100%-category between strata and the prevalences in the 0–49%-category, respectively. We excluded responses from patients not indicating which GP they assessed or assessing non-participating GPs from our analyses.

### Patient characteristics

The patients' age was calculated on the basis of their year of birth and the year of assessment. The patients were grouped into 13 five-year age categories. The patients' level of further education (theoretical education or formalized vocational training after grammar or high school) were categorised as none, less than 2, 2–4 or 5 or more years. Patients undergoing education and patients who could not specify the length of their education formed a separate category. The frequency of attending a GP and the duration of the patient's listing with a particular GP or practice were included as categorical variables (Table 1). Self-rated health was included with its five categories (excellent, very good, good, poor and bad [21]). Patients with chronic conditions were grouped according to the ICPC main category.

**Table 1:** Distribution of characteristics among participating patients.

		**N**	**%**
**Gender**			
N = 27313	Female	18985	69.5
	Male	8328	30.5

**Age **(years)			
N = 27379	18–24	1284	4.7
	25–29	2296	8.4
	30–34	3060	11.2
	35–39	2872	10.5
	40–44	2392	8.7
	45–49	1988	7.3
	50–54	2169	7.9
	55–59	2376	8.7
	60–64	2109	7.7
	65–69	1945	7.1
	70–74	1813	6.6
	75–79	1551	5.7
	80+	1524	5.6

**Further education **(years)			
N = 25870	None	4766	18.4
	Less than 2	2019	7.8
	2–4	14094	54.5
	5 or more	3591	13.9
	Undergoing education or other	1400	5.4

**Frequency of attendance **(last 12 months)			
N = 25860	0–1	2434	9.4
	2–3	7848	30.4
	4–5	6535	25.3
	6–7	3534	13.7
	8–9	1840	7.1
	10+	3669	14.2

**Duration of listing with the GP **(years)			
N = 18134	Less than 1	1431	7.9
	1–2	2065	11.4
	3–7	5600	30.9
	8–12	3962	21.9
	13+	5076	28.0

**Self-rated health**			
N = 27087	Excellent	2350	8.7
	Very good	8121	30.0
	Good	10488	38.7
	Fair	5141	19.0
	Poor	987	3.6

**Chronic condition**			
N = 26801	No	13703	51.1
	Yes-KRTC^1^	4509	16.8
	Yes-other or multiple	8589	32.1

**Chronic condition**			
N = 18212^2^	None	13703	72.1
	K – Cardiovascular	2029	10.7
	R – Respiratory	1095	5.8
	T – Endocrine	1085	5.7
	C – Cancer	300	1.6

^1 ^Reported conditions categorised according to ICPC-2 main categories: K, R and T and the ad hoc-category C (cancer).

^2 ^Patients reporting no chronic condition or reporting KRTC-conditions.

### Statistics

We investigated univariate associations between the patient characteristics and the assessment scores for each of the five dimensions, accounting for the clustering of patients by GPs [22]. Prevalence ratios (PR) with 95%-confidence intervals (95% CI) were preferred to odds ratios (OR) which would tend to overestimate the associations because the prevalence of the variables was high [23,24]. We used generalised linear models (GLM) with log link for Bernoulli family, i.e., modelling the PR. Because of the high prevalence, some of the adjusted GLM analyses could not converge with the Bernoulli family. In these situations we used Poisson regression with robust variance [25]. Furthermore, we adjusted for confounders associated with both assessment and patient characteristics. We found correlations (Pearson's correlation coefficient) between age and frequency of attendance and self-rated health, and accordingly adjusted for these three variables adding gender to the multiple regression analyses. Even though significantly associated with assessment, educational level was not correlated with other patient characteristics and we chose not to adjust for it in the model. Due to relatively high collinearity, we chose to adjust for self-rated health rather than for chronic conditions. In these analyses we also accounted for the clustering of patients. Analyses were performed using complete data only, i.e., the univariate and the GLM analyses were performed using the same data set. We used Stata 9.1 for data processing [26].

#### Ethical approval

Questionnaire surveys such as the present study do not fall within the scope of The Danish National Committee on Biomedical research Ethics. Therefore, we did not need any ethical approval to carry out this study.

#### Informed consent

The participation in this study of both the doctors and the patients was voluntary. The data in this study derive entirely from the evaluation of doctors who had responded to an individual participation to be evaluated by the patients. The patients were asked to answer the questionnaire and as such were free not to do so. The patients knew that aggregated and anonymised replies were fed back to the doctors.

## Results

The GPs distributed a total of 36,561 questionnaires. Valid responses were obtained from 28,260 patients (response rate 77.3%). More than twice as many respondents were female (Table 1) which reflects that women attend a GP twice as often as men [27] and that women are more prone to respond to questionnaires than men (Heje et al., submitted).

Gender differences in assessments (Tables 2, 3, 4, 5, 6 (one table per dimension)) were statistically significant, but numerically quite small. Adjusted analysis showed that male patients assessed "medical care" and "information and support" less favourably, but "organisation of care" more favourably than female patients. In all dimensions the scores increased statistically significantly with increasing patient age. This trend was robust to adjusting. In all dimensions but "medical care", crude PRs for positive evaluations tended to decline with a rising level of education, but this association was eliminated by adjusting.

**Table 2:** Dimension 1: Crude and adjusted associations between patient characteristics and patients' evaluation of aspects of the doctor-patient relationship.

		**100% positive assessments **^**1**^				**0–49% positive assessments **^**2**^			
		
		**Prev.(%)**	**PR**	**Adj. PR**	**95% CI**	**Prev.(%)**	**PR**	**Adj. PR**	**95% CI**
**Gender**									
(N = 25533)	Female	63.8	1	1		14.9	1	1	
	Male	65.1	1.02	0.98	0.96 – 1.00	13.0	*0.87*	0.93	0.86 – 1.00

**Age **(years)									
(N = 25533)	18–24	52.3	1	1		16.4	1	1	
	25–29	54.3	1.04	1.03	0.96 – 1.10	16.6	1.03	1.03	0.87 – 1.22
	30–34	57.6	*1.10*	*1.10*	*1.04 – 1.18*	16.7	1.00	0.99	0.86 – 1.15
	35–39	58.8	*1.12*	*1.15*	*1.08 – 1.22*	17.0	1.01	0.96	0.82 – 1.13
	40–44	60.1	*1.15*	*1.20*	*1.13 – 1.29*	17.3	1.00	0.93	0.80 – 1.09
	45–49	61.2	*1.17*	*1.23*	*1.15 – 1.32*	16.1	*0.90*	*0.83*	*0.70 – 0.98*
	50–54	65.2	*1.25*	*1.33*	*1.25 – 1.42*	14.2	*0.88*	*0.71*	*0.60 – 0.85*
	55–59	67.6	*1.29*	*1.39*	*1.30 – 1.48*	13.0	*0.74*	*0.64*	*0.54 – 0.77*
	60–64	70.2	*1.34*	*1.43*	*1.35 – 1.53*	11.8	*0.67*	*0.58*	*0.48 – 0.70*
	65–69	76.8	*1.47*	*1.57*	*1.47 – 1.67*	9.4	*0.58*	*0.47*	*0.38 – 0.58*
	70–74	76.7	*1.46*	*1.57*	*1.47 – 1.67*	10.5	*0.53*	*0.52*	*0.43 – 0.63*
	75–79	73.6	*1.41*	*1.52*	*1.43 – 1.63*	11.2	*0.51*	*0.55*	*0.44 – 0.67*
	80+	72.8	*1.39*	*1.50*	*1.40 – 1.61*	10.4	*0.57*	*0.49*	*0.39 – 0.62*

**Further education **(years)									
(N = 24364)	None	66.3	1^3^	1^3^		13.3	1	1	
	Less than 2	64.5	*0.94*	1.01	0.97 – 1.05	14.8	1.12	1.05	0.92 – 1.19
	2–4	63.8	*0.86*	0.99	0.96 – 1.02	14.6	1.10	1.07	0.98 – 1.17
	5 or more	62.2	*0.73*	0.96	0.93 – 1.00	14.7	1.10	1.11	0.99 – 1.25
	Undergoing education or other								
		66.0	*0.91*	*1.06*	*1.01 – 1.10*	12.4	0.94	0.91	0.76 – 1.08

**Frequency of attendance **(times per year)									
(N = 25533)	0–1	60.7	1	1		16.4	1	1	
	2–3	61.9	1.02	1.04	1.00 – 1.07	15.7	0.95	0.91	0.82 – 1.01
	4–5	64.6	*1.06*	*1.08*	*1.04 – 1.11*	14.1	*0.86*	*0.79*	*0.71 – 0.88*
	6–7	65.1	*1.07*	*1.10*	*1.05 – 1.14*	14.1	*0.86*	*0.74*	*0.66 – 0.84*
	8–9	67.5	*1.11*	*1.14*	*1.09 – 1.19*	13.0	*0.79*	*0.67*	*0.58 – 0.77*
	10+	68.2	*1.12*	*1.18*	*1.14 – 1.23*	11.4	*0.69*	*0.53*	*0.46 – 0.61*

**Duration of listing **(years)									
(N = 16985)	Less than 1	63.2	1	1		16.5	1	1	
	1–2	64.6	1.02	1.00	0.95 – 1.05	13.6	*0.82*	0.89	0.75 – 1.05
	3–7	64.6	1.02	0.98	0.94 – 1.02	13.7	*0.83*	0.92	0.80 – 1.07
	8–12	65.5	1.04	0.97	0.92 – 1.02	13.4	*0.81*	0.93	0.80 – 1.08
	13+	68.3	*1.08*	1.00	0.95 – 1.05	11.9	*0.72*	*0.85*	*0.73 – 0.99*

**Self-rated health**									
(N = 25533)	Excellent	67.6	1	1		12.0	1	1	
	Very good	64.5	*0.95*	*0.92*	*0.89 – 0.94*	12.5	1.04	*1.14*	*1.01 – 1.29*
	Good	65.0	*0.96*	*0.83*	*0.81 – 0.86*	14.6	*1.22*	*1.66*	*1.46 – 1.87*
	Fair	61.4	*0.91*	*0.74*	*0.72 – 0.77*	17.2	*1.44*	*2.29*	*2.01 – 2.62*
	Poor	58.2	*0.86*	*0.69*	*0.65 – 0.74*	18.6	*1.56*	*2.66*	*2.21 – 3.20*

**Chronic condition**									
(N = 25170**)**	No	62.3	1	1^3^		14.8	1	1	
	Yes-KRTC	70.6	*1.13*	*1.06*	*1.03 – 1.09*	12.2	*0.83*	*0.89*	*0.81 – 0.99*
	Yes-other or multiple	63.9	1.03	*1.04*	*1.01 – 1.06*	14.7	0.99	*0.89*	*0.82 – 0.96*

**Chronic condition**									
(N = 17192)	None	62.3	1	1^3^		14.8	1	1	
	K – Cardiovascular	72.1	*1.16*	*1.05*	*1.01 – 1.09*	10.7	*0.73*	0.85	0.73 – 1.00
	R – Respiratory	66.5	*1.07*	*1.06*	*1.01 – 1.11*	14.9	1.01	0.99	0.85 – 1.15
	T – Endocrine	69.8	*1.12*	1.04	1.00 – 1.09	13.7	0.93	1.01	0.85 – 1.20
	C – Cancer	77.7	*1.25*	*1.20*	*1.12 – 1.28*	6.6	*0.44*	*0.46*	*0.29 – 0.73*

*Prevalence ratio (PR) for 100% positive assessments and for 0–49% positive assessments associated with different patient characteristics. Crude PRs are adjusted for clustering of patients. Adjusted PRs are adjusted for patients' gender, age, frequency of attending a GP, self-rated health and clustering of frequency patients. Numbers in italics are used to indicate statistical significance*.

^1 ^Patients who marked 100% of the answered questions in one of the two most positive answering categories.

^2 ^Patients who marked less than 50% (0–49%) of the answered questions in one of the two most positive answering categories.

^3 ^Poisson regression with robust variance

**Table 3:** Dimension 2: Crude and adjusted associations between patient characteristics and patients' evaluation of aspects of the medical care.

		**100% positive assessments **^**1**^				**0–49% positive assessments **^**2**^			
		
		**Prev.(%)**	**PR**	**Adj. PR**	**95% CI**	**Prev.(%)**	**PR**	**Adj. PR**	**95% CI**
**Gender**									
(N = 25377)	Female	61.2	1	1^3^		19.4	1	1	
	Male	61.2	1.00	*0.95*	*0.93 – 0.97*	19.2	0.99	1.06	1.00 – 1.13

**Age **(years)									
(N = 25377)	18–24	47.8	1	1^3^		26.2	1	1	
	25–29	52.3	*1.09*	1.07	0.99 – 1.16	23.2	1.03	0.92	0.82 – 1.04
	30–34	56.8	*1.19*	*1.18*	*1.10 – 1.26*	21.6	1.00	*0.84*	*0.75 – 0.93*
	35–39	56.3	*1.18*	*1.20*	*1.13 – 1.29*	21.8	1.01	*0.79*	*0.71 – 0.88*
	40–44	57.7	*1.21*	*1.29*	*1.19 – 1.38*	20.9	1.00	*0.71*	*0.63 – 0.79*
	45–49	57.5	*1.20*	*1.31*	*1.22 – 1.41*	21.9	*0.90*	*0.70*	*0.62 – 0.78*
	50–54	61.1	*1.28*	*1.44*	*1.33 – 1.55*	19.0	*0.88*	*0.57*	*0.50 – 0.65*
	55–59	64.5	*1.35*	*1.55*	*1.43 – 1.66*	17.9	*0.74*	*0.52*	*0.46 – 0.60*
	60–64	67.1	*1.40*	*1.61*	*1.50 – 1.73*	15.6	*0.67*	*0.45*	*0.40 – 0.52*
	65–69	73.0	*1.53*	*1.76*	*1.64 – 1.88*	13.1	*0.58*	*0.38*	*0.33 – 0.45*
	70–74	71.4	*1.49*	*1.73*	*1.61 – 1.86*	14.9	*0.53*	*0.43*	*0.38 – 0.50*
	75–79	70.1	*1.47*	*1.73*	*1.61 – 1.85*	14.8	*0.51*	*0.42*	*0.36 – 0.48*
	80+	69.8	*1.46*	*1.73*	*1.60 – 1.87*	15.6	*0.57*	*0.42*	*0.36 – 0.50*

**Further education **(years)									
(N = 24220)	None	60.2	1	1^3^		19.6	1	1	
	Less than 2	60.4	1.01	1.03	0.99 – 1.07	20.8	1.06	1.04	0.94 – 1.16
	2–4	61.5	0.91	1.02	0.99 – 1.05	19.1	0.97	1.02	0.95 – 1.10
	5 or more	61.2	0.80	1.00	0.97 – 1.04	18.8	0.96	1.03	0.93 – 1.14
	Undergoing education or other	61.4	0.90	*1.07*	*1.02 – 1.12*	17.3	0.88	*0.85*	*0.74 – 0.97*

**Frequency of attendance **(times per year)									
(N = 25377)	0–1	64.2	1	1^3^		19.0	1	1	
	2–3	60.9	*0.95*	0.98	0.95 – 1.01	19.2	1.01	0.96	0.88 – 1.05
	4–5	61.9	0.96	1.01	0.98 – 1.05	19.3	1.02	0.92	0.84 – 1.01
	6–7	60.0	*0.94*	1.01	0.98 – 1.06	19.3	1.02	*0.85*	*0.77 – 0.94*
	8–9	60.6	*0.94*	1.04	1.00 – 1.09	19.5	1.03	*0.82*	*0.73 – 0.92*
	10+	60.1	*0.94*	*1.09*	*1.04 – 1.14*	19.8	1.04	*0.74*	*0.67 – 0.83*

**Duration of listing **(years)									
(N = 16880)	Less than 1	60.5	1	1^3^		20.3	1	1	
	1–2	62.7	1.04	1.01	0.96 – 1.07	18.4	0.91	0.99	0.86 – 1.12
	3–7	61.2	1.01	0.96	0.91 – 1.01	19.0	0.93	1.05	0.93 – 1.18
	8–12	62.4	1.03	0.96	0.90 – 1.01	19.1	0.94	1.11	0.98 – 1.27
	13+	65.8	*1.09*	1.00	0.94 – 1.06	16.0	*0.79*	0.94	0.83 – 1.08

**Self-rated health**									
(N = 25377)	Excellent	71.8	1	1^3^		13.4	1	1	
	Very good	64.8	*0.90*	*0.86*	*0.83 – 0.89*	15.7	*1.17*	*1.27*	*1.14 – 1.43*
	Good	61.1	*0.85*	*0.72*	*0.70 – 0.75*	19.2	*1.43*	*1.90*	*1.69 – 2.13*
	Fair	52.4	*0.73*	*0.59*	*0.56 – 0.61*	26.5	*1.97*	*2.94*	*2.60 – 3.31*
	Poor	48.2	*0.67*	*0.53*	*0.49 – 0.58*	30.2	*2.25*	*3.43*	*2.96 – 3.97*

**Chronic condition**									
(N = 25017)	No	64.6	1	1^3^		18.6	1	1	
	Yes-KRTC	67.9	*1.10*	*1.09*	*1.06 – 1.12*	15.9	*0.86*	*0.84*	*0.77 – 0.92*
	Yes-other or multiple	57.1	*0.93*	1.01	0.98 – 1.04	22.2	*1.19*	0.96	0.90 – 1.02

**Chronic condition**									
(N = 17058)		61.6	1	1^3^		18.6	1	1	
	K – Cardiovascular	70.6	*1.15*	*1.12*	*1.08 – 1.16*	13.7	*0.73*	*0.76*	*0.67 – 0.87*
	R – Respiratory	60.6	0.98	1.05	0.99 – 1.10	19.4	1.04	0.90	0.79 – 1.04
	T – Endocrine	68.2	*1.11*	*1.11*	*1.06 – 1.16*	17.9	0.96	0.94	0.81 – 1.08
	C – Cancer	75.2	*1.22*	*1.28*	*1.19 – 1.37*	11.7	*0.63*	*0.55*	*0.39 – 0.77*

*Prevalence ratio (PR) for 100% positive assessments and for 0–49% positive assessments associated with different patient characteristics. Crude PRs are adjusted for clustering of patients. Adjusted PRs are adjusted for patients' gender, age, frequency of attending a GP, self -rated health and clustering of frequency patients. Numbers in italics are used to indicate statistical significance*.

^1 ^Patients who marked 100% of the answered questions in one of the two most positive answering categories.

^2 ^Patients who marked less than 50% (0–49%) of the answered questions in one of the two most positive answering categories.

^3 ^Poisson regression with robust variance

**Table 4:** Dimension 3: Crude and adjusted associations between patient characteristics and patients' evaluation of aspects of the information and support.

		**100% positive assessments **^**1**^				**0–49% positive assessments **^**2**^			
		
		**Prev.(%)**	**PR**	**Adj. PR**	**95% CI**	**Prev.(%)**	**PR**	**Adj. PR**	**95% CI**
**Gender**									
(N = 25154)	Female	64.5	1	1^3^		19.7	1	1	
	Male	64.4	1.00	*0.95*	*0.93 – 0.97*	19.1	0.97	1.05	0.99 – 1.11

**Age **(years)									
(N = 25154)	18–24	52.4	1	1^3^		25.0	1	1	
	25–29	55.5	1.06	1.04	0.97 – 1.12	23.7	1.03	0.96	0.84 – 1.10
	30–34	57.8	*1.10*	*1.10*	*1.03 – 1.18*	22.9	1.00	0.90	0.79 – 1.02
	35–39	60.2	*1.15*	*1.18*	*1.11 – 1.26*	22.5	1.01	*0.84*	*0.74 – 0.94*
	40–44	61.0	*1.16*	*1.23*	*1.15 – 1.32*	22.6	1.00	*0.79*	*0.70 – 0.90*
	45–49	62.1	*1.19*	*1.28*	*1.19 – 1.37*	21.1	*0.90*	*0.71*	*0.61 – 0.81*
	50–54	63.9	*1.22*	*1.34*	*1.26 – 1.43*	18.8	*0.88*	*0.61*	*0.53 – 0.70*
	55–59	68.8	*1.31*	*1.47*	*1.37 – 1.57*	17.2	*0.74*	*0.54*	*0.47 – 0.63*
	60–64	70.9	*1.35*	*1.52*	*1.42 – 1.62*	16.2	*0.67*	*0.51*	*0.44 – 0.59*
	65–69	75.1	*1.43*	*1.61*	*1.50 – 1.72*	13.1	*0.58*	*0.41*	*0.35 – 0.49*
	70–74	75.6	*1.44*	*1.62*	*1.52 – 1.73*	14.4	*0.53*	*0.46*	*0.39 – 0.54*
	75–79	71.7	*1.37*	*1.56*	*1.45 – 1.67*	15.1	*0.51*	*0.47*	*0.40 – 0.56*
	80+	73.7	*1.41*	*1.61*	*1.50 – 1.72*	14.2	*0.57*	*0.43*	*0.36 – 0.51*

**Further education **(years)									
(N = 24003)	None	65.9	1	1^3^		19.1	1	1	
	Less than 2	65.1	*0.95*	1.02	0.98 – 1.06	20.6	1.08	1.03	0.92 – 1.15
	2–4	64.1	*0.85*	0.99	0.96 – 1.01	19.5	1.02	1.01	0.93 – 1.08
	5 or more	63.5	*0.73*	0.97	0.94 – 1.01	19.4	1.01	1.01	0.92 – 1.11
	Undergoing education or other	63.9	*0.84*	1.02	0.97 – 1.07	18.7	0.97	0.91	0.79 – 1.06

**Frequency of attendance **(times per year)									
(N = 25154)	0–1	63.9	1	1^3^		22.5	1	1	
	2–3	63.1	0.99	1.01	0.98 – 1.05	20.8	0.93	*0.89*	*0.81 – 0.98*
	4–5	63.8	1.00	1.03	1.00 – 1.07	18.9	*0.84*	*0.79*	*0.71 – 0.86*
	6–7	65.7	1.03	*1.09*	*1.05 – 1.13*	19.4	*0.86*	*0.75*	*0.68 – 0.83*
	8–9	66.0	1.03	*1.11*	*1.06 – 1.16*	18.5	*0.82*	*0.70*	*0.62 – 0.79*
	10+	66.6	1.04	*1.17*	*1.12 – 1.22*	16.5	*0.73*	*0.57*	*0.51 – 0.63*

**Duration of listing **(years)									
(N = 16745)	Less than 1	63.9	1	1^3^		20.2	1	1	
	1–2	65.4	1.02	0.99	0.94 – 1.05	18.8	0.93	1.01	0.88 – 1.17
	3–7	65.0	1.02	0.97	0.92 – 1.01	18.5	0.91	1.02	0.90 – 1.16
	8–12	66.0	1.03	0.96	0.91 – 1.01	18.2	0.90	1.05	0.91 – 1.21
	13+	68.2	*1.07*	0.98	0.93 – 1.03	17.0	*0.84*	1.01	0.88 – 1.16

**Self-rated health**									
(N = 25154)	Excellent	71.2	1	1^3^		15.7	1	1	
	Very good	66.9	*0.94*	*0.89*	*0.87 – 0.92*	17.2	1.10	*1.21*	*1.09 – 1.35*
	Good	64.5	*0.91*	*0.77*	*0.75 – 0.80*	19.7	*1.26*	*1.72*	*1.55 – 1.91*
	Fair	58.8	*0.83*	*0.66*	*0.63 – 0.68*	23.4	*1.50*	*2.38*	*2.12 – 2.68*
	Poor	55.8	*0.78*	*0.62*	*0.58 – 0.66*	26.5	*1.69*	*2.82*	*2.42 – 3.29*

**Chronic condition**									
(N = 24795)	No	63.7	1	1^3^		20.2	1	1	
	Yes-KRTC	70.9	*1.11*	*1.07*	*1.05 – 1.10*	15.5	*0.77*	*0.82*	*0.75 – 0.89*
	Yes-other or multiple	62.1	0.98	1.02	0.99 – 1.04	20.6	1.02	*0.90*	*0.85 – 0.97*

**Chronic condition**									
(N = 16860)	None	63.7	1	1^3^		20.2	1	1	
	K – Cardiovascular	73.0	*1.15*	*1.09*	*1.05 – 1.13*	14.3	*0.71*	*0.80*	*0.71 – 0.91*
	R – Respiratory	65.5	1.03	*1.06*	*1.01 – 1.11*	19.5	0.97	0.91	0.79 – 1.04
	T – Endocrine	70.7	*1.11*	*1.08*	*1.03 – 1.13*	15.0	*0.74*	*0.79*	*0.68 – 0.92*
	C – Cancer	76.8	*1.21*	*1.21*	*1.13 – 1.31*	11.1	*0.55*	*0.53*	*0.37 – 0.75*

*Prevalence ratio (PR) for 100% positive assessments and for 0–49% positive assessments associated with different patient characteristics. Crude PRs are adjusted for clustering of patients. Adjusted PRs are adjusted for patients' gender, age, frequency of attending a GP, self -rated health and clustering of frequency patients. Numbers in italics are used to indicate statistical significance*.

^1 ^Patients who marked 100% of the answered questions in one of the two most positive answering categories.

^2 ^Patients who marked less than 50% (0–49%) of the answered questions in one of the two most positive answering categories.

^3 ^Poisson regression with robust variance

**Table 5:** Dimension 4: Crude and adjusted associations between patient characteristics and patients' evaluation of aspects of the organisation of care.

		**100% positive assessments **^**1**^				**0–49% positive assessments **^**2**^			
		
		**Prev.(%)**	**PR**	**Adj. PR**	**95% CI**	**Prev.(%)**	**PR**	**Adj. PR**	**95% CI**
**Gender**									
(N = 24154)	Female	64.5	1	1^3^		23.4	1	1	
	Male	68.3	*1.06*	1.01	0.99 – 1.03	*19.4*	*0.83*	*0.91*	*0.85 – 0.96*

**Age **(years)									
(N = 24154)	18–24	58.4	1	1^3^		28.1	1	1	
	25–29	55.8	0.96	0.95	0.89 – 1.01	28.9	1.03	1.04	0.93 – 1.17
	30–34	58.6	1.00	1.00	0.95 – 1.07	27.0	1.00	0.94	0.84 – 1.05
	35–39	60.0	1.03	1.05	0.99 – 1.11	25.2	1.01	*0.85*	*0.76 – 0.95*
	40–44	62.0	1.06	*1.11*	*1.04 – 1.18*	24.3	1.00	*0.78*	*0.69 – 0.89*
	45–49	61.8	1.06	*1.12*	*1.05 – 1.19*	25.3	*0.90*	*0.80*	*0.70 – 0.91*
	50–54	65.5	*1.12*	*1.20*	*1.13 – 1.28*	22.2	*0.88*	*0.68*	*0.60 – 0.78*
	55–59	68.7	*1.18*	*1.27*	*1.20 – 1.35*	20.3	*0.74*	*0.62*	*0.55 – 0.70*
	60–64	71.6	*1.23*	*1.33*	*1.25 – 1.41*	18.0	*0.67*	*0.55*	*0.48 – 0.64*
	65–69	76.3	*1.31*	*1.42*	*1.34 – 1.50*	14.9	*0.58*	*0.46*	*0.39 – 0.53*
	70–74	77.7	*1.33*	*1.44*	*1.36 – 1.53*	14.9	*0.53*	*0.46*	*0.39 – 0.55*
	75–79	76.1	*1.30*	*1.43*	*1.34 – 1.52*	14.5	*0.51*	*0.44*	*0.37 – 0.52*
	80+	76.5	*1.31*	*1.44*	*1.36 – 1.54*	16.3	*0.57*	*0.48*	*0.41 – 0.57*

**Further education **(years)									
(N = 23058)	None	68.5	1	1		20.4	1	1	
	Less than 2	65.1	*0.96*	1.00	0.96 – 1.03	22.2	1.09	1.00	0.90 – 1.11
	2–4	64.6	*0.84*	0.97	0.95 – 1.00	22.8	*1.12*	1.07	0.99 – 1.15
	5 or more	64.8	*0.79*	0.98	0.95 – 1.01	23.0	*1.13*	1.11	1.00 – 1.22
	Undergoing education or other	67.8	*0.85*	*1.05*	*1.01 – 1.10*	20.5	1.00	0.94	0.81 – 1.08

**Frequency of attendance **(times per year)									
(N = 24154)	0–1	66.1	1	1^3^		24.3	1	1	
	2–3	64.3	0.97	0.99	0.96 – 1.03	24.4	1.00	0.96	0.88 – 1.05
	4–5	65.1	0.98	1.01	0.97 – 1.04	22.5	0.93	*0.87*	*0.80 – 0.95*
	6–7	65.7	0.99	1.04	1.00 – 1.08	21.2	*0.87*	*0.78*	*0.70 – 0.86*
	8–9	68.0	1.03	*1.08*	*1.03 – 1.13*	19.4	*0.80*	*0.70*	*0.62 – 0.80*
	10+	68.2	1.03	*1.12*	*1.08 – 1.17*	18.7	*0.77*	*0.63*	*0.57 – 0.69*

**Duration of listing **(years)									
(N = 16168)	Less than 1	65.6	1	1^3^		25.8	1	1	
	1–2	64.8	0.99	0.97	0.92 – 1.02	23.8	0.92	0.97	0.86 – 1.10
	3–7	65.6	1.00	0.96	0.92 – 1.01	21.9	*0.85*	0.92	0.82 – 1.04
	8–12	68.1	1.04	0.98	0.93 – 1.03	20.2	*0.78*	0.89	0.78 – 1.01
	13+	70.7	*1.08*	1.00	0.95 – 1.05	17.5	*0.68*	*0.79*	*0.69 – 0.90*

**Self-rated health**									
(N = 24154)	Excellent	70.0	1	1^3^		19.6	1	1	
	Very good	67.1	*0.96*	*0.92*	*0.89 – 0.95*	21.2	1.08	*1.18*	*1.07 – 1.30*
	Good	66.3	*0.95*	*0.82*	*0.79 – 0.84*	21.8	*1.11*	*1.51*	*1.37 – 1.65*
	Fair	61.1	*0.87*	*0.71*	*0.69 – 0.74*	25.3	*1.29*	*2.02*	*1.82 – 2.24*
	Poor	60.8	*0.87*	*0.70*	*0.65 – 0.74*	25.1	*1.28*	*2.11*	*1.82 – 2.44*

**Chronic condition**									
(N = 23810)	No	64.9	1	1^3^		23.4	1	1	
	Yes-KRTC	71.2	*1.10*	*1.03*	*1.01 – 1.06*	17.9	*0.77*	*0.88*	*0.81 – 0.96*
	Yes-other or multiple	63.8	0.98	1.00	0.98 – 1.03	22.7	0.97	0.93	0.88 – 1.00

**Chronic condition**									
(N = 16049)	None	64.9	1	1^3^		23.4	1	1	
	K – Cardiovascular	66.3	*1.14*	*1.05*	*1.01 – 1.09*	19.8	*0.68*	*0.85*	*0.76 – 0.96*
	R – Respiratory	73.8	1.02	1.02	0.97 – 1.07	16.0	0.95	0.96	0.85 – 1.09
	T – Endocrine	66.2	*1.09*	1.03	0.99 – 1.08	22.1	0.81	0.92	0.80 – 1.07
	C – Cancer	70.5	*1.16*	*1.12*	*1.04 – 1.21*	18.8	0.56	*0.62*	*0.46 – 0.84*

*Prevalence ratio (PR) for 100% positive assessments and for 0–49% positive assessments associated with different patient characteristics. Crude PRs are adjusted for clustering of patients. Adjusted PRs are adjusted for patients' gender, age, frequency of attending a GP, self-rated health and clustering of patients. Numbers in italics are used to indicate statistical significance*.

^1 ^Patients who marked 100% of the answered questions in one of the two most positive answering categories.

^2 ^Patients who marked less than 50% (0–49%) of the answered questions in one of the two most positive answering categories.

^3 ^Poisson regression with robust variance

**Table 6:** Dimension 5: Crude and adjusted associations between patient characteristics and patients' evaluation of aspects of the accessibility.

		**100% positive assessments **^**1**^				**0–49% positive assessments **^**2**^			
		
		**Prev.(%)**	**PR**	**Adj. PR**	**95% CI**	**Prev.(%)**	**PR**	**Adj.PR**	**95% CI**
**Gender**									
(N = 25560)	Female	29.1	1	1		34.0	1	1	
	Male	32.3	*1.11*	0.97	0.93 – 1.02	30.7	*0.90*	0.99	0.94 – 1.03

**Age **(years)									
(N = 25560)	18–24	17.8	1	1		39.5	1	1	
	25–29	19.7	1.11	1.09	0.94 – 1.26	40.7	1.03	1.04	0.96 – 1.13
	30–34	21.0	*1.18*	*1.19*	*1.03 – 1.37*	39.4	1.00	0.98	0.90 – 1.07
	35–39	21.5	*1.21*	*1.25*	*1.09 – 1.44*	40.0	1.01	0.97	0.89 – 1.06
	40–44	23.6	*1.32*	*1.42*	*1.23 – 1.64*	39.5	1.00	0.93	0.85 – 1.02
	45–49	26.5	*1.49*	*1.62*	*1.41 – 1.87*	35.7	*0.90*	*0.83*	*0.76 – 0.91*
	50–54	27.9	*1.57*	*1.75*	*1.52 – 2.01*	35.0	*0.88*	*0.80*	*0.73 – 0.88*
	55–59	34.3	*1.92*	*2.17*	*1.90 – 2.48*	29.4	*0.74*	*0.67*	*0.61 – 0.75*
	60–64	38.5	*2.16*	*2.44*	*2.12 – 2.80*	26.5	*0.67*	*0.60*	*0.54 – 0.67*
	65–69	42.6	*2.39*	*2.72*	*2.37 – 3.12*	22.9	*0.58*	*0.53*	*0.46 – 0.59*
	70–74	46.7	*2.62*	*2.99*	*2.61 – 3.42*	20.8	*0.53*	*0.48*	*0.42 – 0.54*
	75–79	46.5	*2.61*	*2.97*	*2.60 – 3.39*	20.2	*0.51*	*0.46*	*0.40 – 0.53*
	80+	45.7	*2.56*	*2.96*	*2.57 – 3.41*	22.4	*0.57*	*0.51*	*0.45 – 0.57*

**Further education **(years)									
(N = 24385)	None	32.6	1	1		31.7	1	1	
	Less than 2	30.6	*0.90*	1.08	0.99 – 1.16	34.7	*1.09*	1.00	0.93 – 1.08
	2–4	28.6	*0.76*	1.00	0.95 – 1.06	34.1	*1.08*	0.98	0.93 – 1.03
	5 or more	29.3	*0.66*	1.02	0.94 – 1.10	32.1	1.01	0.93	0.86 – 1.01
	Undergoing education or other	32.2	*0.80*	*1.16*	*1.06 – 1.27*	28.8	0.91	*0.83*	*0.75 – 0.91*

**Frequency of attendance **(times per year)									
(N = 25560)	0–1	28.3	1	1		35.9	1	1	
	2–3	26.9	0.95	0.99	0.92 – 1.07	37.0	1.03	1.00	0.94 – 1.06
	4–5	29.8	1.05	1.06	0.98 – 1.14	33.5	*0.93*	*0.92*	*0.86 – 0.98*
	6–7	31.4	*1.11*	*1.14*	*1.04 – 1.24*	30.0	*0.84*	*0.81*	*0.75 – 0.87*
	8–9	33.9	*1.20*	*1.21*	*1.11 – 1.33*	28.5	*0.79*	*0.77*	*0.70 – 0.84*
	10+	35.3	*1.25*	*1.30*	*1.20 – 1.42*	26.8	*0.75*	*0.69*	*0.64 – 0.75*

**Duration of listing **(years)									
(N = 17001)	Less than 1	24.6	1	1		36.8	1	1	
	1–2	25.9	1.05	0.99	0.87 – 1.12	36.1	0.98	1.03	0.94 – 1.12
	3–7	27.2	1.11	1.00	0.89 – 1.12	34.7	0.94	1.01	0.93 – 1.09
	8–12	31.8	*1.29*	1.12	0.98 – 1.28	31.6	*0.86*	0.95	0.86 – 1.05
	13+	38.0	*1.55*	*1.29*	*1.13 – 1.48*	26.5	*0.72*	*0.82*	*0.73 – 0.92*

**Self-rated health**									
(N = 25560)	Excellent	30.6	1	1		30.1	1	1	
	Very good	28.6	0.93	*0.83*	*0.78 – 0.89*	33.6	*1.11*	*1.19*	*1.11 – 1.28*
	Good	30.6	1.00	*0.68*	*0.64 – 0.73*	33.4	*1.11*	*1.44*	*1.34 – 1.54*
	Fair	30.7	1.00	*0.60*	*0.55 – 0.65*	33.3	*1.11*	*1.64*	*1.51 – 1.78*
	Poor	33.0	1.08	*0.62*	*0.56 – 0.70*	29.2	0.97	*1.49*	*1.31 – 1.69*

**Chronic condition**									
(N = 25197)	No	27.6	1	1		35.2	1	1	
	Yes-KRTC	36.6	*1.33*	1.04	0.98 – 1.10	27.9	*0.79*	0.97	0.91 – 1.03
	Yes-other or multiple	30.5	*1.10*	1.01	0.96 – 1.06	32.1	*0.91*	0.95	0.91 – 1.00

**Chronic condition**									
(N = 17208)	None	27.6	1	1		35.2	1	1	
	K – Cardiovascular	37.7	*1.37*	1.01	0.94 – 1.09	26.1	*0.74*	0.96	0.86 – 1.06
	R – Respiratory	32.3	*1.17*	1.04	0.95 – 1.15	31.8	0.90	0.99	0.90 – 1.09
	T – Endocrine	37.0	*1.34*	1.08	0.99 – 1.17	28.9	*0.82*	0.98	0.88 – 1.09
	C – Cancer	42.7	*1.55*	*1.15*	*1.01 – 1.32*	22.3	*0.63*	*0.78*	*0.61 – 0.98*

*Prevalence ratio (PR) for 100% positive assessments and for 0–49% positive assessments associated with different patient characteristics. Crude PRs are adjusted for clustering of patients. Adjusted PRs are adjusted for patients' gender, age, frequency of attending a GP, self-rated health and clustering of patients. Numbers in italics are used to indicate statistical significance*.

^1 ^Patients who marked 100% of the answered questions in one of the two most positive answering categories.

^2 ^Patients who marked less than 50% (0–49%) of the answered questions in one of the two most positive answering categories.

^3 ^Poisson regression with robust variance

Scores for all dimensions rose with an increasing frequency of attendance. Adjustment for patient characteristics eliminated this association for "medical care", but not for the other dimension. Scores also tended to increase the longer time the patients had been listed with the GP, but not after adjusting for confounding patient characteristics.

We found consistent, statistically significantly decreasing scores with decreasing self-rated health in all dimensions which was even more pronounced after controlling for confounders.

Patients reporting a chronic condition gave more positive assessments of their GP – the most positive being the patients with "KRTC-conditions". The associations were modified but not eliminated upon adjustment except for "accessibility" where we found no association.

## Discussion

We found a positive GP assessment to be associated with increasing patient age and increasing frequency of attendance. Patients reporting a chronic condition were more positive, whereas a poor self-rated health was strongly associated with less positive scores also after adjustment. The association between patient gender and assessment was weak and inconsistent and depended on the focus. We found no association either with the patients' educational level or with the duration of listing with the GP even after adjusting for patient characteristics.

This project was part of a larger national patient evaluation project, which may have introduced some sources of bias. Thus, all GPs in the involved counties were invited and those who signed in may not necessarily be a representative sample. The method for patient inclusion would ideally secure a random sample of the doctor-seeking part of the listed patients where frequently attending patients, evidently, would be overrepresented. We do not know to what extent GPs forgot to hand out questionnaires or even if they more systematically let some patients out. However, in this study we focused on adjusted associations between assessments and patient characteristics. Selection bias would therefore seem to have a smaller impact than if we had studied actual levels of assessment.

Duration of further education is only a rough indicator of education. We chose this simple indicator because it lends itself better to use in a self-administered questionnaire than more complex indicators. A certain recall bias may have affected indications of frequency of attendance and duration of listing with the GP. Such information bias may be differentiated which would tend to overestimate the magnitude, but not the direction of the associations found [24].

We learned from our pilot-study that many patients did not understand the expression "chronic illness", so we added "or a serious disease lasting more than three months". This probably enhanced the sensitivity of the question but lowered its specificity, resulting in overrepresentation of more trivial conditions. In order to be able to compare evaluations by patients with a genuine chronic disease with that of those with no chronic conditions, we divided the respondents into three groups: those reporting no chronic condition, those reporting a cardiovascular, respiratory, endocrine or cancer diagnosis (ICPC-2-categories K, R and T and the ad hoc category C), and those reporting other or multiple diagnoses. This may have resulted in the exclusion of some very ill patients with for instance cardiovascular disease in addition to diabetes thus tending to underestimate the significance of suffering from a chronic condition.

This study enjoyed a very high statistical power with over 27,000 cases included. We were therefore able to detect quite small, statistically significant associations. Some statistically significant associations were so small that their clinical relevance could be questioned. However, the considerable power of our analyses is accompanied by an almost negligent risk of overlooking associations (type II-error).

While some earlier studies have presented diverging results on the association between patient gender and assessment [6,13,28], we found only small and inconsistent associations, which is in concordance with a meta-analysis performed by Hall et al. [11]. This finding may be rooted in the absence of any gender influence on the way patients experience health care or in the GPs' possible intuitive adjustment of their care to the different needs of different patients [9].

The adjusted analyses showed a strong positive association between patient age and assessment level, which is also a consistent finding in other studies [6,11]. This association may be rooted not only in the long-standing relationship with their GP and a higher age-related morbidity, but also in a more realistic view on health, health care expectancies and doctors' skills due to the patients' life experience. The finding may also be due to a general positivity of – some may say more mellow way of judging by – older people [29]. However, we could also be facing to a cohort-effect which, though, is less probable considering the linearity of the association.

Crude analysis showed an expected negative association between educational level and assessment scores, which is in concordance with earlier findings [11]. In our study, however, the association was eliminated after adjustment except for the heterogenous group of patients undergoing education and patients who were unable to report the length of their education, which indicates that the association may have been confounded by other characteristics. This difference may be due to the use of different methods for measuring educational level, but it may also reflect that associations found in one cultural setting may not necessarily be valid in another.

Frequency of attendance is a multifaceted variable. For example, we do not know if a high number of encounters is the result of the patient's or the GP's initiative (ex. half-yearly control-appointments for chronic disease). Still, it is an indicator of the intensity of the doctor-patient relationship, just as the duration of listing with the GP is an indicator of the relational continuity between the GP and his patient [30]. Patients' age, time on the GP's list, frequency of attendance and health are closely interconnected. Our adjusted PRs therefore capture a more "clean" effect on the assessment of being listed for years with the same GP and of the frequency of attending the GP. Adjustment of the latter for health ensured that the positive assessment was not an expression of the ill patient relying on the quality of the GP care [28].

Continuity is one of the core qualities of the doctor-patient-relationship in a health care system where the GP is the patient's primary contact with the health care system [31]. The possible migration of dissatisfied patients from the GPs' lists favours a positive association between assessment and time on the GPs list. However, unlike Hjortdahl and Laerum [32] we found no association with the duration of the relationship but a positive association with the intensity. This may indicate that the positive association between continuity and assessment demonstrated in earlier studies [10,33,34] may be correlated with other characteristics which we adjusted for in the present study.

We found diverging results regarding the association between health and assessment of the care depending on whether we looked at self rated health or diagnosed chronic illness. In a paper by Rahmqvist [14] this was very well illustrated. The health indicator used in this study was a mix of self and physician ratings and the study found no association between the patients' health and their rating of the care. Hall et al. [35] also used a mixed health indicator with a seeming emphasis on self rated health indicators and found that poor health was associated with dissatisfaction.

Both Hall et al. [36,37] and Wensing et al. [38] found an association between less positive assessments and poor self-rated health. We also found this strong negative association between self-rated health and assessment after adjusting for confounders, but we also found that patients who reported a chronic condition assessed more positively in all dimensions except accessibility with patients suffering from cardiovascular, respiratory, endocrine and cancer diseases giving the most positive assessments. This is an assessment paradox because GPs received more negative assessments from patients with a poor self-rated health, but more positive assessments from patients with chronic illness. This was also found by Zapka et al. [39]. This may be due to our adjusting for self-rated health which may be somewhat risky when dealing with chronic illness. On the other hand, a possible explanation may be that the GPs were more capable at handling patients with exact diagnoses and maybe even capable of improving their self-rated health, than at handling patients who rated their health as poor did not fit into a specific disease category – e.g. patients with somatization disorders [40]. We may have been demonstrating an effect of the clinical recommendations on the handling of different chronic diseases that have been implemented in Danish general practice through the past few years. All in all, these results illustrates how the use of different health indicators may affect the association between health and assessment and that it is crucial to specify how health is measured whenever this parameter is being used.

We only included a limited number of patient variables. Inclusion of more and specific variables reflecting psycho-socio-cultural aspects might have added value to the study; in particular, it might have helped explain the oppositely directed associations between self-rated health, chronic conditions and assessments.

If patients' demands, expectations and experience, which are determinants of satisfaction in most models [6,7], were always in balance we would probably see no assessment variation between patients with different characteristics. But, as we have demonstrated, assessments do vary with patient characteristics. In this paper we chose to publish the crude as well as the adjusted results of the analyses of possible associations between patient characteristics and evaluations. This kind of results serves to point out possible quality deficits in general, and to serve this purpose the results need to be adjusted for possible confounders. However, our study offers no possibility for deciding whether the source for evaluation differences between groups of patients is embedded with the patient or with the care and hence with the GP.

Whether or not the results from patient evaluations of care providers should be adjusted for uneven distribution of patient characteristics also depends on the purpose for which they are produced. Adjustment for patient differences may produce a more fair comparison between GPs, and when patient evaluations are used for accreditation purposes it may also seem fair to adjust for differences in the evaluated GPs' patient populations. Yet, adjustment may also blur the assessment of GPs' ability to meet the needs of the populations actually served [9,11] and thus render quality improvement at a GP level difficult.

## Conclusion

In a setting with a comprehensive list system and gatekeeping, we performed a patient evaluation study using a validated international questionnaire among voluntarily participating GPs and a large number of patients, thus producing results with high statistical precision. After adjusting for patients' gender, age, frequency of attending a GP and self-rated health we confirmed findings from earlier studies that there is a positive association between patients' age and frequency of attendance and their assessment of their GP and a weak and inconsistent association with patients' gender. We also showed that patients reporting a chronic condition were more positive in their assessment of the GP than patients without a chronic condition, whereas in the same population assessing the same aspects of practice, the assessments turned out less positive with decreasing self-rated health. We were not able to demonstrate associations with patients' level of education or the time the patients had been listed with the GP.

In this study we demonstrated statistically significant but yet minor associations between patients' characteristics and their assessment of the GP. Some of the variation may also be associated with GP and practice characteristics as we have demonstrated in a related already published paper [41]. The results from this study may lead to further investigations into the causes behind the found associations and to improving activities in case they are due to quality deficits. We will leave it to the relevant parties to discuss and decide whether or not they should be used for standardising future quality assessments for comparison purposes.

## Competing interests

The authors declare that they have no competing interests.

## Authors' contributions

HNH, PV and FO planned the study, and HNH carried out the patient evaluation survey assisted by the research secretariat. IS, PV and HNH planned the statistical analyses, which were performed by IS. HNH drafted the manuscript, which was rewritten by HNH, PV, FO and IS. All authors read and approved the final manuscript

## Pre-publication history

The pre-publication history for this paper can be accessed here:



## Supplementary Material

Additional file 1 The EUROPEP-questionnaire. Click here for file

Additional file 2 Danish general practice. Click here for file
