# Supplementary material for: Patient characteristics associated with differences in patients' evaluation of their general practitioner
Source: BMC Health Serv Res. 2008 Aug 20;8:178. doi: 10.1186/1472-6963-8-178 (PMC2533311; doi:10.1186/1472-6963-8-178)
Supplement: Additional file 2 — Danish general practice. [file 1472-6963-8-178-S2.pdf]

Additional file 2
Danish general practice

Denmark (5.5 mio. inhabitants) is divided into 16 health administrative regions with 50,000 to 625,000 inhabitants. In each region the Public Health Insurance regulates the number of general practitioners. 98% of the inhabitants are registered (listed) with a local GP and receive tax-supported free medical care. The GPs are self-employed, but are paid by the Public Health Insurance both per capita (inhabitants on the GP's list) and on a fee for service basis (70%/30%). The GPs serve as gatekeepers vis-à-vis the rest of the health care system (i.e. access to a hospital or specialist care implies referral by a GP) and are responsible for the care of all registered patients 24 hours a day. The GPs in a region co-operate about the out-of-hours care.

The GPs can organise their practice in several ways: A practice can be owned by one GP – a **single-handed practice**. A single-handed practice can be owned by two GPs who are working part-time – a **shared single-handed** practice. In this case the Public Health Insurance has decided that the patient list size must not exceed that of a standard single-handed practice. A **group of single-handed** GPs can share premises and staff, but not patients. A group of GPs can also own a larger practice together and serve a list of patients matching the number of GPs – a **partnership practice**. If the GPs in a partnership outnumber the list of patients in terms of patients per GP, they usually work part-time – a **part-time partnership.** In this case the Public Health Insurance also sets limits for the list size.
